# Internal validation and evaluation of the predictive performance of models based on the PRISM-3 (Pediatric Risk of Mortality) and PIM-3 (Pediatric Index of Mortality) scoring systems for predicting mortality in Pediatric Intensive Care Units (PICUs)

**DOI:** 10.1186/s12887-022-03228-y

**Published:** 2022-04-12

**Authors:** Zahra Rahmatinejad, Fatemeh Rahmatinejad, Majid Sezavar, Fariba Tohidinezhad, Ameen Abu-Hanna, Saeid Eslami

**Affiliations:** 1 Department of Medical Informatics, Faculty of Medicine, Mashhad University of Medical Sciences, Mashhad, Iran; 2 Department of Medical Records and Health Information Technology, School of Paramedical Sciences, Mashhad University of Medical Sciences, Mashhad, Iran; 3 Pediatric Intensive Care, Department of Pediatrics, Faculty of Medicine, Mashhad University of Medical Sciences, Mashhad, Iran; 4 Department of Medical Informatics, Amsterdam UMC - Location AMC, University of Amsterdam, Amsterdam, the Netherlands

**Keywords:** Scoring system, PRISM, PIM, Mortality

## Abstract

**Purpose:**

The study was aimed to assess the prognostic power The Pediatric Risk of Mortality-3 (PRISM-3) and the Pediatric Index of Mortality-3 (PIM-3) to predict in-hospital mortality in a sample of patients admitted to the PICUs.

**Design and methods:**

The study was performed to include all children younger than 18 years of age admitted to receive critical care in two hospitals, Mashhad, northeast of Iran from December 2017 to November 2018. The predictive performance was quantified in terms of the overall performance by measuring the Brier Score (BS) and standardized mortality ratio (SMR), discrimination by assessing the AUC, and calibration by applying the Hosmer-Lemeshow test.

**Results:**

A total of 2446 patients with the median age of 4.2 months (56% male) were included in the study. The PICU and in-hospital mortality were 12.4 and 16.14%, respectively. The BS of the PRISM-3 and PIM-3 was 0.088 and 0.093 for PICU mortality and 0.108 and 0.113 for in-hospital mortality. For the entire sample, the SMR of the PRISM-3 and PIM-3 were 1.34 and 1.37 for PICU mortality and 1.73 and 1.78 for in-hospital mortality, respectively. The PRISM-3 demonstrated significantly higher discrimination power in comparison with the PIM-3 (AUC = 0.829 vs 0.745) for in-hospital mortality. (AUC = 0.779 vs 0.739) for in-hospital mortality. The HL test revealed poor calibration for both models in both outcomes.

**Conclusions:**

The performance measures of PRISM-3 were better than PIM-3 in both PICU and in-hospital mortality. However, further recalibration and modification studies are required to improve the predictive power to a clinically acceptable level before daily clinical use.

**Practice implications:**

The calibration of the PRISM-3 model is more satisfactory than PIM-3, however both models have fair discrimination power.

## Introduction

Recent advances in therapeutic protocols and medical facilities highlight the need for accurate prediction systems [1]. Such risk prediction models can be used in tasks such as benchmarking for the evaluation the effectiveness and efficiency of pediatric intensive care (PICUs), early detection of critically ill patients, and optimizing resource allocation which may result in better quality of care and patient safety, especially in low and middle-income countries [2]. These countries including Iran have scarce resources, especially when a surge of critically ill pediatric patients leads to disproportionate disbalance between needs and available resources.

In this circumstance, employing an accurate, well-validated, and easy-to-calculate risk assessment instrument can benefit prioritizing patients and optimizing resource use in the PICUs [3, 4]. Such risk assessment instruments can be based on scoring systems, which use the worst physiological and laboratory values during the first 12–24 h of admission to indicate severity of illness. A higher score represents higher severity [5].

Multiple scoring systems have been introduced, some of which are widely used to predict the risk of death in children such as the Pediatric Risk of Mortality (PRISM) and the Pediatric Index of Mortality (PIM). The PRISM was developed using the data collected from 11,165 patients admitted to PICUs in the USA [6] whereas, the PIM was developed based on the data of ICUs located in the UK, Australia, Ireland, and New Zealand [7]. The third version of these scoring systems (PRISM-3 and PIM-3) is commonly used in the Intensive Care Units (ICUs) for years after its introduction [8]. Over the last decade, there have been significant advances in pediatric intensive care among developing countries. However, in countries with low- and middle-income as well as with a higher pediatric population, there is still a need to PICUs, a greater number of competent health care professionals, timely access to required medicine, and equipment to successfully contribute to the reduction of pediatric mortality. The predictive performance of models based on the PRISM-3 and PIM-3 scores for PICU mortality and in-hospital mortality are not well understood, especially in developing countries including our country. Hence, this study is aimed at evaluating and comparing the predictive performance of prediction models based on the PRISM-3 and PIM-3 scores in a sample of patients admitted to the PICU a developing country [9]. Hence, this study is aimed at evaluating and comparing the predictive performance of prediction models based on the PRISM-3 and PIM-3 scores in a sample of patients admitted to the PICU a developing country.

## Method

### Study design and setting

We designed a multicenter, retrospective cohort study of severely ill children admitting to the six tertiary PICUs at two university hospitals for a period of 12 months, from December 2017 to November 2018, in Mashhad, northeast of Iran. Each hospital had an average of 1500 admissions per year and each PICU was equipped with an average of 7.2 beds.

Both centers are general pediatric hospitals and admit all sorts of cases (medical and surgical). However according to the subspecialty approach of the hospitals, the majority of oncology, nephrology and hematology cases were treated in “hospital A” and many cases of surgical, rheumatology, lung, infectious, gastrointestinal and neurological were treated to “hospital B”. The PICUs in each hospital do not differ in terms of the type of patients referred, but since smaller beds are intended for younger patients, the only difference is related to age classification.

### Study population

All children (i.e., aged younger than 18 years) admitted to the PICU were eligible in the study. We excluded from the analysis patients with brain death at the time of admission, and patients who stayed in PICU for less than 2 h and discharge or expire before 24 h of admission. In addition, those patients who were referred to subspecialty hospitals are also excluded. Individuals with missing values for the main variables which is essential for calculating the scores were imputed using the chained equations approach implemented in the mice package available in R. It should be noted that re-admissions due to different diagnoses were considered as new admissions.

### Study variables

The following variables were collected: age, gender, diagnosis, other main variables for calculating the scores, as well as length of stay (LOS) at both PICU and hospital, and the two outcome variables PICU mortality and in-hospital mortality.

The key variables were collected to calculate the PRISM-3 score: arterial blood gas, glucose, creatinine, Glasgow Coma Score (GCS), respiratory rate, systolic and diastolic blood pressure, heart rate, pupillary reactions to bright light, blood urea, potassium, platelet, white blood cell count temperature, Prothrombin Time (PT), and Partial Thromboplastin Time (PTT) [5]. The variables used to calculate the PIM-3 score were as follows: Low, high, or very high-risk diagnosis. Low-risk diagnosis including: asthma, bronchiolitis, croup, obstructive sleep apnea, diabetic, ketoacidosis, and seizure. High-risk diagnosis including: spontaneous cerebral hemorrhage, cardiomyopathy or myocarditis, hypoplastic left heart syndrome, neurodegenerative disorder, necrotizing enterocolitis. Very high-risk diagnosis including: cardiac arrest, severe combined immune deficiency, leukemia or lymphoma, bone marrow transplant recipient, and liver failure.

systolic blood pressure, base excess, type of admission (Emergency, referral, and elective), FiO2, PaO_2_, mechanical ventilation support, recovery from surgery as the main reason for admission to the PICU, and admission due to cardiac bypass [10]. The other vital signs and.

Since these two models categorized the age variable according to the month. We also considered the month as a unit of age (see Table 1 which summarizes these models in terms of variables, unit of variables, the formula for calculating and their point assignment schemes). It should be noted that all of the units associated with each variable is provided in Tables 2 and 3. The unit for the LOS was consider day.

**Table 1:** The point assignment scheme of each scoring system

	Cardiovascular and Neurologic Vital Signs					
**PRISM-3 score**	**Age Range:** Neonate 0 to < 1 month Infant 1 to 12 month Child > 12 to 144 months Adolescent > 144 months	SBP (mm Hg) Neonate > 55 mmHg →0 Neonate 40–55 mmHg →3 Neonate < 40 mmHg →7 Infant > 65 mmHg →0 Infant 45–65 mmHg →3 Infant < 45 mmHg →7 Child > 75 mmHg →0 Child 55–75 mmHg →3 Child < 55 mmHg →7 Adolescent > 85 mmHg →0 Adolescent 65–85 mmHg →3 Adolescent < 65 mmHg →7	Heart Rate (beats/minute) Neonate < 215 →0 Neonate 215–225 →3 Neonate > 225 →4 Infant < 215 →0 Infant 215–225 →3 Infant > 225 →4 Child < 185 →0 Child 185–205 →3 Child > 205 →4 Adolescent < 145 →0 Adolescent 145–155 →3 Adolescent > 155 →4	Temp (°C) < 33→ 3 33–40 →0 > 40→ 3	**Mental status** GCS ≥ 8→ 0 GCS < 8 →5	**Pupillary response** Both reactive →0 1 reactive (1 fixed and > 3 mm) →7 Both fixed and both > 3 mm→11
	**Blood Gasometery**					
	Acidosis pH > 7.28 & TCO2 ≥ 17 mEq/L →0 pH 7.0–7.28 or TCO2 5–16.9 mEq/L →2 pH < 7.0 or TCO2 < 5 mEq/L →6		**PCO2** (mm Hg) < 50 →0 50–75→ 1 > 75 →3	PH < 7.48 →0 7.48–7.55 →2 > 7.55 →3	**TCO2**(mEq/L) ≤ 34 →0 > 34 →4	**PaO2** (mmHg) ≥ 50 →0 42–49.9 →3 < 42 → 6
	**Chemistry Tests**					
	Creatin (mg/dL) Neonate ≤0.85 →0 Neonate > 0.85 →2 Infant ≤0.90 →0 Infant > 0.90 →2 Child ≤0.90 →0 Child > 0.90 →2 Adolescent ≤1.30→0 Adolescent > 1.30 →2		Potassium (mEq/L) ≤ 6.9 →0 > 6.9 →3	Glucose (mg/dL) ≤ 200 →0 > 200 →2		BUN (mg/dL) Neonate ≤11.9 →0 Neonate > 11.9 →3 Not neonate ≤14.9 →0 Not neonate > 14.9 →3
	**Hematologic Tests**					
	PT & PTT Neonate PT ≤ 22 s and PTT ≤ 85 s →0 Neonate PT > 22 s or PTT > 85 s →3 Not neonate PT ≤ 22 s & PTT ≤ 57 s →0 Not neonate PT > 22 s or PTT > 57 s →3		Platelet (μL) > 200,000 →0 100,000–200,000 →2 50,000-99,999→ 4 < 50,000 →5	WBC (μL) ≥ 3000 →0 < 3000 →4		
	Logit = 0.207 × PRISM-3 score - (0.005 × (age in months) – 0.433 × 1 (if postoperative) – 4.782					
**PIM-3 score**	PIM-3 score = (3.8233 × pupillary reaction) + (− 0.5378 × elective admission) + (0.9763 × mechanical ventilation) + (0.0671 × [absolute {base excess}]) + (− 0.0431 × SBP) + (0.1716 × [SBP2/1000]) + (0.4214 × [{FiO2 × 100}/PaO2]) − (1.2246 × bypass cardiac procedure) − (0.8762 × non-bypass cardiac procedure) − (1.5164 × noncardiac procedure) + (1.6225 × very high-risk diagnosis) + (1.0725 × high-risk diagnosis) − (2.1766 × low-risk diagnosis) − 1.7928.					
	Probability of death = exp. (PIM-3 score)/[1 + exp. (PIM-3 score)]					

*Abbreviation*: *SBP* systolic blood pressure, *Temp* temperature, *PCO2* partial pressure of carbon dioxide, *PH* potential of hydrogen, *TCO2* total carbon dioxide, *PaO2* partial pressure of oxygen, *WBC* White blood cells, *PT* Prothrombin Time, *PTT* Partial Thromboplastin Time, *PIM-3* Pediatric Index of Mortality version 3, *PRISM-3* Pediatric Risk of mortality. Low-risk diagnosis including: asthma, bronchiolitis, croup, obstructive sleep apnea, diabetic, ketoacidosis, and seizure. High-risk diagnosis including: spontaneous cerebral hemorrhage, cardiomyopathy or myocarditis, hypoplastic left heart syndrome, neurodegenerative disorder, necrotizing enterocolitis. Very high-risk diagnosis including: cardiac arrest, severe combined immune deficiency, leukemia or lymphoma, bone marrow transplant recipient, and liver failure

**Table 2:** Baseline demographic and clinical characteristics of the patients admitted to the PICU with available and missing data

Characteristics		PICU mortality		*P*-Value	In-hospital mortality		*P*-Value	Missing data	Number of Missing
		Non-survive ***N*** = 304	Survive ***N*** = 2142		Non-Survive ***N*** = 395	Survive ***N*** = 2051		(*N* = 338)	
Demographics									
Age (Month)		5.9(0.67–49.4)	4(0.67–22)	0.013 ^a^	6.4(0.9–51)	3.93(0.63–21.17)	< 0.001 ^a^	3.6 (0.27–18.5)	0
Neonate (< 1 month)		82(27.0%)	604(28.2%)	0.023 ^c^	101(14.8%)	585(85.2%)	< 0.001 ^c^	100(29.6%)	0
Infant (1–12 month)		94(30.9%)	824(38.3%)		125(13.7%)	790(86.3%)		136(40.2%)	0
Child (12–144 month)		117(38.5%)	675(31.5%)		150(19%)	642(81%)		98(29%)	0
Adolescent (> 144 month)		11(3.6%)	42(2.0%)		19(4.8%)	34(1.7%)		4(1.2%)	0
Male		161(53.0%)	1218(56.9%)	0.20 ^c^	209(9%)	1170(47%)	0.13 ^c^	197(58.3)	0
Female		143(47.0%)	924(43.1%)		186(7.4%)	881(36.6%)		141(41.7)	0
Vital signs									
Temperature(°C)		37(36.5–37.5)	37(36–37)	0.015 ^a^	37(36–37.5)	37(36.8–37.4)	0.14 ^a^	37(36–37.4)	60
Diastolic (mmHg)		56(40–70)	55(44–68)	0.682 ^a^	56(40–69)	55(44–68)	0.561 ^a^	55(43–69)	42
Systolic (mmHg)		95(73.2–111.8)	97(81–110)	0.038 ^a^	95(75–111)	97(81–110)	< 0.001 ^a^	98(79–109)	42
Fio2(mmHg)		40(29–90)	21(21–40)	< 0.001 ^a^	40(29–80)	21(21–40)	< 0.001 ^a^	21(21–40)	0
RR (breaths/min)		38(28–44)	38(28–44)	0.114 ^a^	35(25–45)	37(28–44)	0.274 ^a^	35(25–42)	35
Pupillary response	Normal	232(76.3%)	2075(96.9%)	< 0.001^b^	1996(81.6%)	311(12.7%)	< 0.001^b^	321(95.5%)	0
	Abnormal	72(23.7%)	67(3.1%)		55(2.3%)	84(3.4%)		15(4.5%)	0
Lab result tests									
Glucose (mg/dL)		124.5(92–178)	110(87–143)	< 0.001 ^a^	122(90–170)	110(87–143)	0.001 ^a^	93(71–129.7)	0
Urea (mg/dL)		32.8(19–51)	21(14–33)	< 0.001 ^a^	30(18–48)	21(14–33)	< 0.001 ^a^	20(14–30)	0
Cr (mg/dL)		0.90(0.5–1)	0.5(0.5–0.7)	< 0.001 ^a^	0.85(0.5–0.9)	0.78(0.5–0.7)	< 0.001 ^a^	0.58(0.5–0.71)	0
K (mEq/L)		4.3(3.5–4.9)	4.4(3.9–4.8)	0.022 ^a^	4.3(3.5–4.9)	4.4(3.9–4.8)	0.003 ^a^	4.22(3.7–4.7)	48
GCS		12(7–15)	15(13–15)	< 0.001 ^a^	12(7–15)	15(13–15)	< 0.001 ^a^	15(13–15)	71
Plate let (10^3^ cells/mm3)		155(56.5–273)	296(202–419)	< 0.001 ^a^	159(56–278)	300(207–421)	< 0.001 ^a^	291(183–432)	89
PT		14.5(12.6–20.7)	13(12–14)	< 0.001 ^a^	14(12.5–19.4)	13(12–14)	< 0.001 ^a^	13(12–14)	97
PTT		36(30–48)	32(29–36)	< 0.001 ^a^	35(30–45)	32(29–36)	< 0.001 ^a^	32(30–37)	65
HR (beats/min)		139(118–156.7)	136(120–153)	0.455 ^a^	138(119–156)	136(120–153)	0.427 ^a^	133(120–151)	42
WBC (10^3^ cells/mm3)		11.7(6.4–17.3)	8.3(11.6–16.1)	0.394 ^a^	11.4(6.1–17.0)	11.7 (8.4–16.1)	0.041 ^a^	11(8.5–15.8)	89
PCO2(mmHg)		32(26.5–39.7)	32.4(26.6–39)	0.480 ^a^	35(27–42)	32(26–38)	0.775 ^a^	36.9(30.1–43.4)	18
PH		7.34(7.24–7.41)	7.37(7.30–7.41)	< 0.001 ^a^	7.34(7.26–7.41)	7.37(7.30–7.41)	< 0.001 ^a^	7.34(7.29–7.39)	18
TCO2(mEq/L)		20.4(16.1–23.6)	21(18–236)	0.06 ^a^	21(16.5–24.2)	21(18–23.5)	0.384 ^a^	21.34(19–24.3)	18
PaO2(mmHg)		94(92–99)	95.9(95–99)	0.004 ^a^	93.93(92–99)	96.04(94–100)	< 0.001 ^a^	97(95–99)	18
Readmission		79(26%)	523(24.4%)	0.298 ^b^	105(26.6%)	497(24.2%)	0.176 ^b^	138(40.8%)	0
Type admission									
Emergency		108(35.3%)	618(28.9%)	0.008 ^c^	584(25%)	142(5%)	0.001 ^c^	133(39.3%)	0
Referral		142(46.7%)	1035(48.3%)		995(41%)	182(7%)		171(50.6%)	0
Elective		54(17.8%)	489(22.8%)		472(20%)	71(2%)		34(10.1%)	0
Type of diagnosis based on PIM-3									
Very High Risk		104(34.2%)	98(4.5%)	< 0.001 ^c^	127(32.2%)	75(3.7%)	< 0.001 ^c^	11(3.3%)	0
High Risk		42(13.8%)	46(2.1%)		53(13.4%)	35(1.7%)		3(0.9%)	0
Low Risk		36(11.8%)	174(8.1%)		51(12.9%)	159(7.8%)		5(1.5%)	0
Mechanical ventilation		70(23%)	159(7.45%)	< 0.001 ^b^	89(3.7%)	140(5.7%)	< 0.001^b^	39(11.5%)	0
PRISM-3 score		12(8,19)	5(2,8.2)	< 0.001 ^a^	11(7,19)	5(2,8)	< 0.001 ^a^	5(2–9)	0
PIM-3 score		4(2,9)	2.(2,3)	< 0.001 ^a^	4(2,8)	2.(2,3)	< 0.001 ^a^	2(2–4)	0
LOS in Hospital (day)		11(5–23.7)	8(4–15)	< 0.001 ^a^	12(5–26)	8(4–15)	< 0.001 ^a^	9(4–17)	0
LOS in ICU (day)		8(3–18.5)	7(3.7–13)	< 0.029 ^a^	8(3–19)	7(4–12)	0.004 ^a^	7(4–14)	0
Type of diagnosis based on the ICD-10									
Congenital malformation		62(20.4%)	539(25.2%)	0.365 ^c^	88(22.3%)	513(85.3%)	0.266 ^c^	114(33.7%)	0
Diseases of the digestive system		43(14.1%)	296(13.8%)		54(13.7%)	285(84.0%)		63(18.6%)	0
Diseases of the respiratory system		42(13.8%)	224(10.5%)		51(12.9%)	215(80.8%)		31(9.2) %	0
Neoplasms, Diseases of the blood		34(11.2%)	167(7.8%)		43(10.9%)	158(78.6%)		17(5.1%)	0
Diseases of genitourinary system		15(4.9%)	94(4.4%)		23(5.82%)	86(78.8%)		11 (3.3%)	0
Infectious diseases		15(4.9%)	115(5.4%)		22(5.6%)	108(83%)		8 (2.4%)	0
Metabolic diseases		15(4.9%)	102(4.8%)		18(4.6%)	99(84.6%)		12 (3.6%)	0
Diseases of the circulatory system		10(3.3%)	73(3.4%)		14(3.5%)	69(83.1%)		10 (3%)	0
Diseases of the nervous system		8(2.6%)	55(2.6%)		8(2%)	55(87.3%)		7(2.1%)	0
Certain conditions originating in the perinatal period		6(2%)	61(2.8%)		7(1.8%)	60(89.6%)		15(4.4%)	0
Other disease		54(17.8%)	416(19.3%)		67(16.9%)	403(85.8%)		50(14.7%)	0

Values represented as median (IQR)

*Abbreviations*: *Cr* creatinine; potassium, *GCS* Glasgow coma scale, *PT* Prothrombin Time, *PTT* Partial Thromboplastin Time, *HR* Heart Rate, *WBC* White Blood Cell, *PCO*_*2*_ Partial pressure of carbon dioxide, *TCO2* Total Carbon Dioxide, *PaO2* partial pressure of oxygen, *FiO2* Fraction of inspired oxygen, *RR* Respiratory Rate, *ICD10* International Statistical Classification of Diseases and Related Health Problems 10th Revision

^a^ Analysis by Mann-Whitney U. ^b^ Analysis by Fisher’s exact test. ^c^ Analysis by Chi-square test

**Table 3:** Baseline demographic and clinical characteristics of the patients admitted to the PICU after imputation

Characteristics		PICU mortality		*P*-Value	In-hospital mortality		*P*-Value
		Non-survive ***N*** = 338	Survive ***N*** = 2446		Non-Survive ***N*** = 434	Survive ***N*** = 2350	
Demographics							
Age (month)		5.1(0.49–42.7)	3.98(0.63–21.4)	0.031 ^a^	5.7(0.7–47.8)	3.9(0.6–20.7)	< 0.001 ^a^
Neonate (< 1 month)		95(28.1%)	691(28.2%)	0.016 ^c^	115(26.4%)	671(28.6%)	< 0.001 ^c^
Infant (1–12 month)		106(31.4%)	944(38.6%)		138(31.8%)	912(38.8%)	
Child (12–144 month)		126(37.3%)	765(31.3%)		162(37.4%)	729(31.0%)	
Adolescent (> 144 month)		11(3.2%)	46(1.9%)		19(4.4%)	38(1.6%)	
Male		181(53.5%)	1393(57%)	0.242 ^c^	232(53.5%)	1342(57.1%)	0.155 ^c^
Female		157(46.5%)	1053(43%)		202(46.5%)	1008(42.9%)	
Vital signs							
Temperature(°C)		37(36.5–37.5)	37(36.8–37.4)	0.008 ^a^	37(36–37.5)	37(36.8–37.4)	0.086 ^a^
Diastolic (mmHg)		56(40–69)	55(44–68)	0.512 ^a^	56(40–69)	55(44–68)	0.440 ^a^
Systolic (mmHg)		95(72–110)	97(81–110)	0.006 ^a^	95(73–110)	97(81–110)	0.009 ^a^
Fio2(mmHg)		40(21–90)	21(21–40)	< 0.001 ^a^	40(28–80)	21(21–40)	< 0.001 ^a^
RR (breaths/min)		35(25–44)	37(28–43)	0.109 ^a^	35(25–44)	37(28–43)	0.287 ^a^
Pupillary response	Normal	260 (9.9%)	2370(90.1%)	< 0.001^c^	343(13.1%)	2287(86.9%)	< 0.001^c^
	Abnormal	78(50.6%)	76(49.4%)		91(59.1%)	63(40.9%)	
Lab result tests							
Glucose (mg/dL)		122.0(90–176)	108(85–142)	< 0.001 ^a^	120(90–167)	108(85–142)	< 0.001 ^a^
Urea (mg/dL)		31.9(19–49.9)	21(14–33)	< 0.001 ^a^	30(18–48)	21(14–31)	< 0.001 ^a^
Cr (mg/dL)		0.76(0.5–0.7)	0.88(0.5–1)	< 0.001 ^a^	0.76(0.5–0.7)	0.84(0.5–0.9)	< 0.001 ^a^
K (mEq/L)		4.3(3.5–4.8)	4.4(3.8–4.8)	0.020 ^a^	4.2(3.5–4.8)	4.4(3.9–4.8)	0.002 ^a^
GCS		12(7–15)	15(13–15)	< 0.001 ^a^	12(7–15)	15(13–15)	< 0.001 ^a^
Platelet (10^3^ cells/mm3)		158(61–273)	298(202–420.7)	< 0.001 ^a^	163(61–277.5)	301(206–425)	< 0.001 ^a^
PT		14.5(12.7–20.2)	13(12–14)	< 0.001 ^a^	14(12.5–19.2)	13(12–14)	< 0.001 ^a^
PTT		36(30–47)	32(29–36)	< 0.001 ^a^	35(30–45)	32(29–36)	< 0.001 ^a^
HR (beats/min)		138(117–157)	136(120–152)	0.411 ^a^	138(119–156)	136(120–153)	0.539 ^a^
WBC (10^3^ cells/mm3)		11.9(6.5–17.3)	11.6(8.4–16)	0.63 ^a^	11.5(6.1–17.0)	11.6 (8.4–16.0)	0.08 ^a^
PCO_2_(mmHg)		33.8(27.3–40.1)	32.9(26.9–39.7)	0.165 ^a^	33.3(27.4–39.7)	33(26.9–39.8)	0.608 ^a^
PH		7.34(7.23–7.41)	7.36(7.30–7.41)	< 0.001^a^	7.34(7.25–7.41)	7.36(7.30–7.41)	< 0.001 ^a^
TCO_2_(mEq/L)		20.6(16.4–24.0)	21(18.2–23.6)	0.098 ^a^	21(16.6–24.4)	21(18.3–23.6)	0.386 ^a^
PaO2(mmHg)		94(92–99)	95.9(95–99)	0.004 ^a^	94(92–99)	96(94–100)	< 0.001 ^a^
Readmission		1.66 ± 1.55	1.54 ± 1.17	0.001 ^a^	1.65 ± 1.5	1.54 ± 1.17	< 0.001 ^a^
Type admission							
Emergency		120(35.5%)	738(30.2%)	0.001 ^c^	156(36.0%)	702(29.9%)	0.029 ^c^
Referral		158(46.7%)	1191(48.7%)		201(46.3%)	1148(48.9%)	
Elective		60(17.8%)	517(21.1%)		77(17.7%)	500(21.2%)	
Type of diagnosis based on PIM-3							
Very High Risk		111(32.8%)	102(4.17%)	< 0.001 ^c^	134(30.9%)	79(2.6%)	< 0.001 ^c^
High Risk		23(6.8%)	43(1.75%)		30(7%)	36(1.5%)	
Low Risk		23(6.8%)	164(6.7%)		33(7.6%)	154(6.6%)	
Mechanical ventilation		70(20.7%)	159(6.5%)	< 0.001 ^b^	101(23.3%)	165(7%)	< 0.001^c^
PRISM-3 score		12(8,19)	5(2,8.2)	< 0.001 ^a^	11(7,18)	5(2,8)	< 0.001 ^a^
PIM-3 score		4(2,8)	2(2,3)	< 0.001 ^a^	4(2,8)	2(2,3)	< 0.001 ^a^
LOS in Hospital (day)		12(5–23)	8(4–15)	< 0.001 ^a^	12(5–25.2)	8(4–15)	< 0.001 ^a^
LOS in ICU (day)		8(3–17)	7(4–13)	< 0.008 ^a^	8(3–19)	7(4–13)	0.001 ^a^
Type of diagnosis based on the ICD-10							
Congenital malformation		69(20.5%)	646(26.4%)	0.079 ^c^	97(22.4%)	618(26.3%)	0.071 ^c^
Diseases of the digestive system		50 (14.8%)	353(14.4%)		62(14.3%)	341(14.5%)	
Diseases of the respiratory system		45(13.3%)	252(10.3%)		55(12.7%)	242(10.3%)	
Neoplasms, Diseases of the blood		41(12.1%)	178(7.2%)		50(11.5%)	169(7.2%)	
Diseases of genitourinary system		16(4.7%)	104(4.2%)		24(5.5%)	96 (4.1%)	
Infectious diseases		15(4.4%)	123(5.0%)		22(5.1%)	116(4.9%)	
Metabolic diseases		16(4.7%)	113(4.6%)		20(4.6%)	109(4.6%)	
Diseases of the circulatory system		10(3.0%)	83(3.4%)		14(3.2%)	79(3.4%)	
Diseases of the nervous system		9(2.7%)	61(2.5%)		9(2.1%)	61(2.6%)	
Certain conditions originating in the perinatal period		9(2.7%)	74(3.0%)		10(2.3%)	73(3.1%)	
Other disease		58(17.1%)	459(19%)		71(16.3%)	446(19%)	

Values represented as median (IQR)

*Abbreviations*: *Cr* creatinine; potassium, *GCS* Glasgow coma scale, *PT* Prothrombin Time, *PTT* Partial Thromboplastin Time, *HR* Heart Rate, *WBC* White Blood Cell, *PCO*_*2*_ Partial pressure of carbon dioxide, *TCO2* Total Carbon Dioxide, *PaO2* partial pressure of oxygen, *FiO2* Fraction of inspired oxygen, *RR* Respiratory Rate, *ICD10* International Statistical Classification of Diseases and Related Health Problems 10th Revision

^a^ Analysis by Mann-Whitney U. ^b^ Analysis by Fisher’s exact test. ^c^ Analysis by Chi-square test

### Statistical analyses

Normality of continuous variables was assessed via the ShapiroWilk test. The data did not follow a normal distribution, so we compared the groups by utilizing nonparametric techniques. The Mann-Whitney U test was used for comparisons of continuous variables between survivors and non-survivors. The Chi-square test or Fisher’s exact test were also used to compare categorical data. Data were presented as median (IQR) for continuous variables and as the frequency (%) for categorical variables.

The PIM-3 and PRISM-3 sores were calculated retrospectively by the researchers for each patient based on the measurements at the time of admission to the PICU. The formula for calculating PRISM-3 and PIM-3 score is presented in Table 1.

After calculating the scores of each scoring system, we applied logistic regression analysis to predict both PICU and in-hospital mortality as response variable by using the PRISM-3 and PIM-3 scores as the explanatory variables, separately. The logit formula was used to calculate the probability of mortality as following:

$$\mathrm{P}=\frac{1}{1+\exp \left[-\left({\beta}_0+{\beta}_1X\right)\right]}$$

(β_0_: Intercept; β_1_: Coefficient of the score; X: score)

Then the predictive performance of the models was assessed in terms of the overall accuracy, discrimination, and calibration. The discrimination ability of the probabilistic models as measured by the AUC is exactly the same as the discrimination ability of the original scores they are based on. This is because the model keeps the same order in the probabilities as in the scores (i.e. if we sort the probabilities in ascending order it will result in the same order as with the score). The probabilistic model however allows us to investigate the additional performance measures of calibration and the Brier score.

The predictive performance of the models was quantified with respect to the accuracy of the predicted probabilities, discrimination, and calibration. The accuracy between the predicted and observed probabilities was assessed by the Brier Score (BS), which is the mean squared difference between the observed and predicted outcome and using a standardized mortality ratio (SMR), which is the ratio of the risk-adjusted observed mortality to the expected mortality derived from the development set where the score was developed. Discrimination between survivors and non-survivors was quantified by the Area Under the Receiver Operating Characteristic Curve (AUC). Calibration, which is a measure of the agreement between the predicted and observed probabilities was assessed by calibration and lack of agreement was tested by the Hosmer-Lemshow. Moreover, the Negative Predictive Value (NPV), Positive Predictive Value (PPV), specificity, and sensitivity were calculated using the Youden Index threshold [11]. We used bootstrapping with 1000 samples to internally validate the model and calculate the bias-corrected estimate of the AUC and its confidence intervals (CI) and the Delong’s method was used to compare the two AUCs. Statistical significance was set at the 0.05 *p*-value level. All analyses were performed using the R statistical environment (with packages rms, Hmisc, pROC, and mice).

## Results

**Fig. 1:**
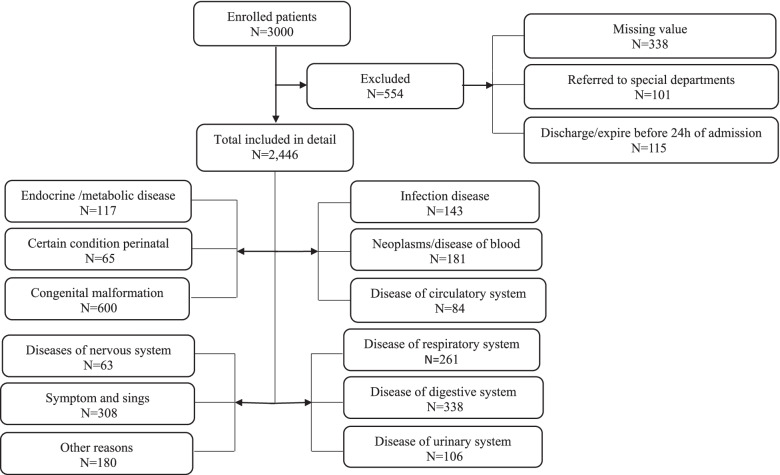
The flowchart diagram of the patient inclusion process

In total, 3000 patients were eligible and met the inclusion criteria. After applying the exclusion criteria, 2784 patients remained for further analyses (Fig. 1). The data had about 11.3% of missing values, which were imputed as described in the Statistical Analysis section.

The PICU and in-hospital mortality were 12.14 and 15.58%, respectively. Table 2 and Table 3 demonstrate the baseline characteristics of the study population before and after imputation. The median length of both the PICU and hospital stay were 7(3–13) and 8(4.7–16), respectively (See Table 2 and Table 3).

A total of 1379 (56.4%) patients were male and the median age of the patients was 4.2 months (IQR: 0.66–24) the majority of the patients were younger than 12 months (65.43%). Generally, of the demographic profile, age was associated with outcome (*p* < 0.001) while gender did not show any significant influence on the outcome (*p* = 0.13).

The congenital malformation, digestive system disease, and patient with the respiratory diseases accounted for 24.5, 13.8, and 10.6% of the admissions, respectively. The cause of mortality according to the ICD-10 coding system were as follows: 88 (22.3%) congenital malformations, 54 (13.7%) digestive system diseases, 51 (12.9%) the respiratory diseases, 43 (10. 9%) blood and neoplasm diseases, 23 (5.82%) kidney diseases, 22 (5.6%) infectious diseases, 18 (4.6%) metabolic diseases, 14 (3.5%) cardiac disease, 8 (2%) neurological disorders, 7 (1.8%) perinatal diseases, and 67 (16.9%) other diagnostic groups.

The mean score of PRISM-3 and PIM-3 were 6.9 ± 6.5 and 3 ± 2.8 respectively. About 48% of patient were referral cases, 30% were brought in by emergency medical services, and 9.4% of patients required mechanical ventilation support.

As shown in Tables 2, 3 and Table 4, predominantly those patients in the age group of 12–144 months had the worst outcome, and this pattern is similar in both PICU (19%) and in-hospital mortality (38.5%). These patients were mainly assigned the diagnosis belonging to neoplasm, circulatory, respiratory, and also digestive system categories.

**Table 4:** Predictive characteristics of PRISM-3 and PIM-3 to predict PICU and in-hospital mortality

Outcome	Age group	MODEL	AUC 95%CI	Quintiles of risk	Total	Exp	Obs	SMR	HL	BS	Cut-off	Sen	Spe	PPV	NPV	Acc
**PICU mortality**	Total	PRISM-3	0.831(0.814–0.854)	1:PRISM-3	687	6.97	2	1.34	< 0.001	0.088	6.**5**	0.881	0.632	0.253	0.974	0.663
		PIM-3	0.745(0.722–0.781)	2:PRISM-3	333	15.01	14		0.014	0.093	2.5	0.700	0.666	0.229	0.940	0.670
	Neonate	PRISM-3	0.841(0.813–0.868)	3:PRISM-3	506	40.31	40		0.118	0.087	6.5	0.878	0.620	0.239	0.974	0.651
		PIM-3	0.752(0.710–0.794)	4:PRISM-3	484	60.39	90		0.037	0.091	3.5	0.500	0.837	0.294	0.925	0.797
	Infant	PRISM-3	0.855(0.819–0.891)	5:PRISM-3	436	104.5	158		0.003	0.073	6.5	0.893	0.693	0.250	0.982	0.713
		PIM-3	0.804(0.752–0.856)	1:PIM-3	391	0	9	1.37	0.071	0.076	4.5	0.595	0.883	0.368	0.950	0.853
	Child	PRISM-3	0.811(0.774–0.849)	2:PIM-3	1127	65.98	82		< 0.001	0.102	7.5	0.846	0.654	0.298	0.960	0.683
		PIM-3	0.736(0.686–0.786)	3:PIM-3	231	21.26	30		0.003	0.107	2.5	0.658	0.675	0.260	0.919	0.672
	Adolescent	PRISM-3	0.697(0.523–0.871)	4:PIM-3	405	52.39	60		0.927	0.144	11.5	0.727	0.574	0.285	0.90	0.603
		PIM-3	0.775(0.614–0.936)	5:PIM-3	292	82.18	123		0.861	0.141	3.5	0.818	0.574	0.310	0.931	0.620
	Hospital A with 1380 patients: AUC for PRISM-3: 0.839(0.808–0.869) with 0.15 Standard Error & AUC for PIM-3: 0.744(0.697–0.790) with 0.24 Standard Error Hospital B with 1404 patients: AUC for PRISM-3: 0.821(0.793–0.850) with 0.14 Standard Error & AUC for PIM-3: 0.743(0.703–0.783) with 0.20 Standard Error															
**In-hospital mortality**	Total	PRISM-3	0.781 (0.759–0.808)	1:PRISM-3	687	6.97	30	1.73	0.068	0.108	6.5	0.785	0.636	0.294	0.939	0.660
		PIM-3	0.737 (0.718–0.771)	2:PRISM-3	333	15.01	28		0.026	0.113	2.5	0.689	0.680	0.293	0.919	0.681
	Neonate	PRISM-3	0.777(0.727–0.827)	3:PRISM-3	506	40.31	49		0.765	0.104	9.5	0.614	0.807	0.354	0.924	0.778
		PIM-3	0.753(0.701–0.805)	4:PRISM-3	484	60.39	97		0.183	0.102	2.5	0.644	0.762	0.319	0.925	0.745
	Infant	PRISM-3	0.768(0.719–0.816)	5:PRISM-3	436	104	191		0.299	0.095	6.5	0.744	0.692	0.277	0.945	0.699
		PIM-3	0.761(0.711–0.811)	1:PIM-3	391	0	16	1.78	0.237	0.099	3.5	0.672	0.725	0.279	0.933	0.718
	Child	PRISM-3	0.775(0.733–0.816)	2:PIM-3	1127	65.98	107		0.034	0.125	7.5	0.773	0.663	0.349	0.926	0.684
		PIM-3	0.726(0.680–0.772)	3:PIM-3	231	21.26	40		0.130	0.129	2.5	0.646	0.690	0.327	0.893	0.681
	Adolescent	PRISM-3	0.822(0.701–0.942)	4:PIM-3	405	52.39	88		0.876	0.188	14	0.684	0.746	0.565	0.828	0.724
		PIM-3	0.840(0.726–0.953)	5:PIM-3	292	82.18	144		0.486	0.197	3.5	0.842	0.666	0.551	0.896	0.724
	Hospital A with 1380 patients: AUC for PRISM-3: 0.784 (0.747–0.821) with 0.19 Standard Error & AUC for PIM-3: 0.725(0.681–0.769) with 0.23 Standard Error Hospital B with 1404 patients: AUC for PRISM-3: 0.773(0.740–0.805) with 0.17 Standard Error & AUC for PIM-3: 0.740 (0.705–0.775) with 0.18 Standard Error															

*Abbreviations*: *SMR* standardized mortality ratio, *Exp* expected mortality, *Obs* observed mortality, *HL* Hosmer–Lemeshow, *BS* Brier Score, *Sen* Sensitivity, *Spe* Septicity, *PPV* Positive predictive value, *NPV* Negative predictive value, *Acc* Accuracy, *PRISM* Pediatric Risk of Mortality, *PIM* Pediatric Index of Mortality

The linear predictors of the logistic regression models presented per outcome, separately, are:

For predicting PICU mortality:

$$\mathrm{PRISM}-3:-3.056+0.174\times \mathrm{PRISM}-3\_\mathrm{score},\mathrm{and}\ \mathrm{PIM}-3:-3.075+0.297\times \mathrm{PIM}-3\_\mathrm{score}.$$

For predicting in-hospital mortality:

$$\mathrm{PRISM}-3:-3.094+0.166\times \mathrm{PRISM}-3\_\mathrm{score},\mathrm{and}\ \mathrm{PIM}-3:-2.772+0.312\times \mathrm{PIM}-3\_\mathrm{score}.$$

**Fig. 2:**
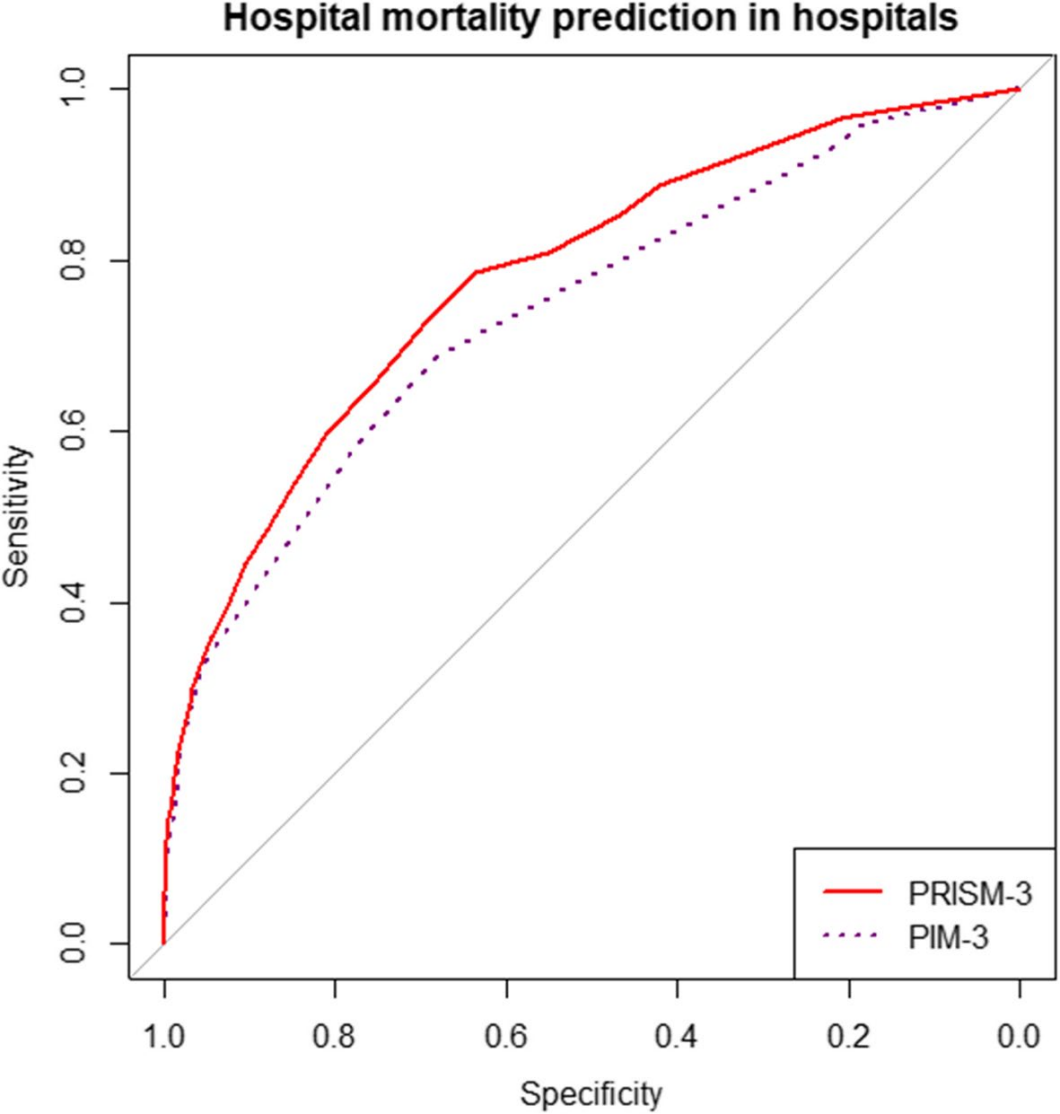
Receiver operating characteristic curve of the PRISM-3 and PIM-3 in hospitals

**Fig. 3:**
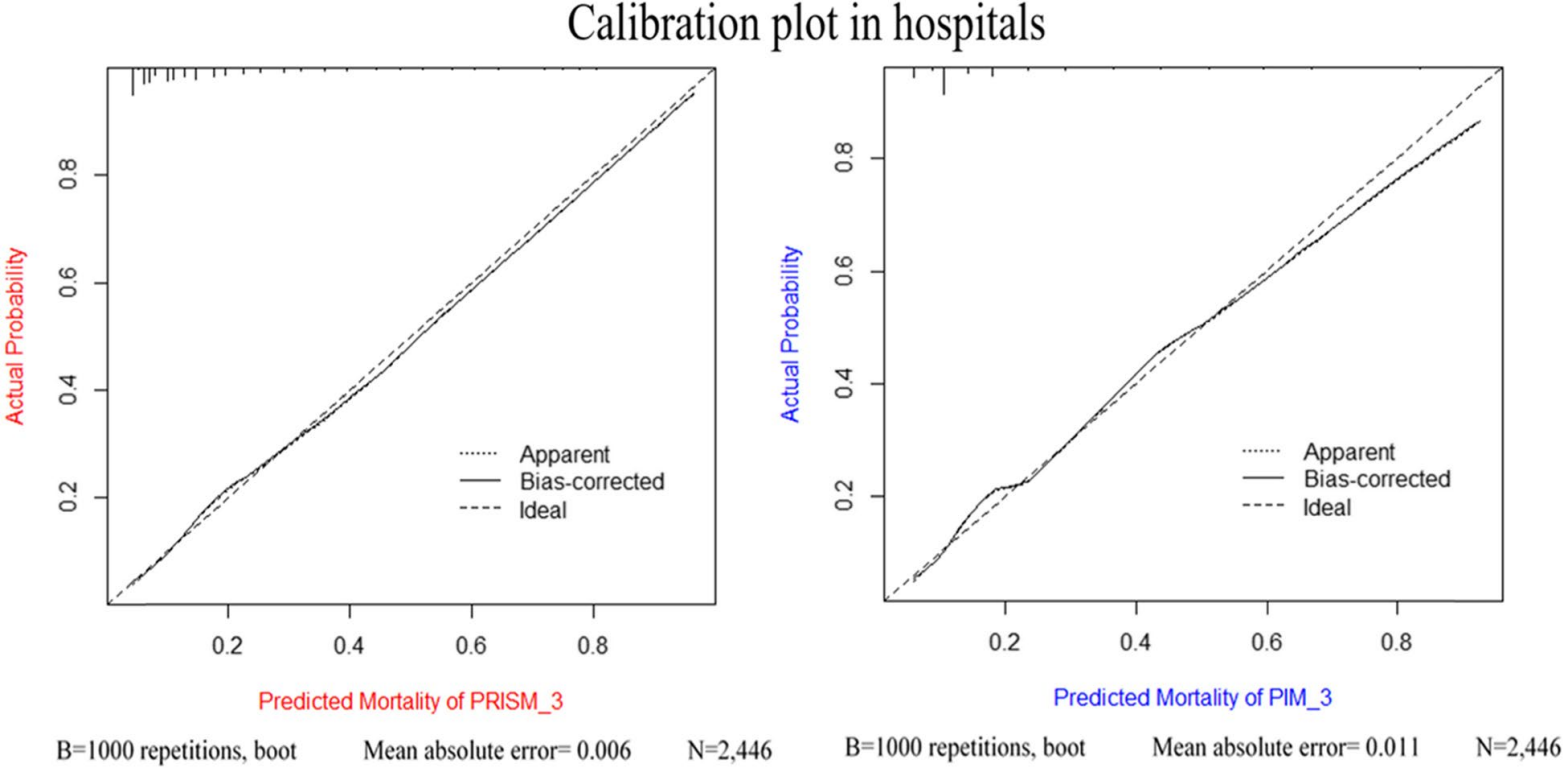
Calibration curves for the observed mortality against predicted risk of death for PIM-3 and PRISM-3 models in hospitals

The BS, SMR, AUC, HL-test, and other characteristics of both models for PICU and in-hospital mortality prediction, as well as according to age groups are presented in Table 4. The BS of the PRISM-3 and PIM-3 was 0.088 and 0.093 for PICU mortality and 0.108 and 0.113 for in-hospital mortality. The SMR of the PRISM-3 and PIM-3 was 1.34 (CI 95%: 1.19–1.49) and 1.37 (CI 95%: 1.21–1.52) for PICU mortality and 1.73 (CI 95%: 1.56–1.90) and 1.78 (CI 95%: 1.6–1.95) for in-hospital mortality, respectively. The PRISM-3 demonstrated significantly higher discrimination power in comparison with the PIM-3 (AUC = 0.831 vs 0.745) for in-hospital mortality and (AUC = 0.781 vs 0.737) for in-hospital mortality. The HL test revealed poor calibration for both models in both outcomes. The difference in the AUCs for PRISM-3 and PIM-3 models are significantly significant (*P* = 0.001) (see Fig. 2 and Table 4). The calibration graphs of both models are shown in Fig. 3.

## Discussion

### Main findings

This multi-center study aimed to evaluate the performance of the models based on the PRISM-3 and PIM-3 scores in predicting both PICU and in-hospital mortality. We found that the overall performance of PRISM-3 and PIM-3 were comparable for in-hospital mortality in terms of the Brier score. The discrimination power of PRISM-3, however, was significantly higher than the PIM-3 for both PICU and in-hospital mortality. Interestingly, when considering PICU mortality as an outcome, the PRISM-3 appears to be much more discriminative (AUC: 0.78 vs 0.83). A possible explanation is that predicting a short-term outcome is easier than a longer-term outcome. With the exception of the adolescent age group, the PRISM-3 was far superior in predicting PICU and hospital mortality than PIM-3.

The models were not well calibrated in predicting PICU mortality nor in-hospital morality. One possible explanation is that the original models were developed for western populations and are now being applied to an Asian population in a developing country. Generally, with respect to the discrimination ability, the PRISM-3 performed significantly better than the PIM-3. A possible explanation for this is the consideration of more important factors. However, PRISM-3 requires the collection of 17 variables while the PIM-3 requires the collection of only 12 variables which makes the former a more demanding model [11]. Practicality, just as clinical sensibility, may play an important role in clinical applications. Generally, the purpose of designing a prediction model is to offer a reliable model that can be transported and used in clinical practice; hence, it is critical to choose a model that is reasonably simple but does not sacrifice substantial predictive performance. The requirement for a succinct decision method may be even more important in the PICUs in developing countries, where clinicians frequently deal with complicated and severely ill children as well as limited resources. Having an objective method, using either the more complex model if applicable, or the simpler model if that is opportune, can assist them in prioritizing and managing complex patients, as well as enhancing benchmarking indices. Our findings reveal that the PICU and in-hospital mortality were 12.4 and 16.4%, respectively. The PICU mortality in our study is much higher than in European and the US PICUs (12.4 vs 2.5) [12, 13]. However, the PICU mortality rate in our study is situated in the middle of the mortality range in developing countries (range from 8.4% for Korea to 40% for Egypt). There are various reasons for the discrepancy in mortality rate between our study and in western countries. To begin with, the two centers in our study are referral hospitals so they frequently deal with the most critically ill patients. In addition, the disease profile in the present study is also different from studies in western countries. For instance, the majority of patients have also suffered from congenital malformation, digestive and respiratory and cancer disease, and treatments were more challenging for these patients. Furthermore, the other explanation of higher mortality in our study compared to developed countries is the difference in quality and standards of care, equipment used, and the relatively undeveloped medical care level. So, these differences necessitate a significant effort for improvement.

In comparison to previous investigations that have been conducted in Iran, the Middle East, and Asia, this is one of the largest studies that examine the prognostic performance of the PRISM-3 and PIM-3 in predicting pediatric patient outcomes (both PICU and in-hospital mortality). All of those studies were performed in Iran were single center and the median sample size was only 221 (min-max: 90–365) tend to be located at higher frequencies in male gender and the majority of the patients were included samples was younger than 40 months. These investigations determined that the PRISM-3 differential power was between the range of fair (AUC:0.70–0.80) to adequate (AUC:0.80–0.90).

In general, with respect to Table 5, most of related studies have been performed on small samples, the median AUCs for the PIM-3 and PRISM-3 in similar studies were 0.82 [min-max: 0.72–0.89] and 0.82 [min-max: 0.56–0.93], respectively [2, 12, 16, 17, 21, 29, 31, 33–35]. In most of the studies, the AUC of the PRISM-3 was higher than PIM-3. A study found that the AUC of PRISM-3 was significantly higher than the PIM-3 (*P* = 0.04) [21], which is in line with our findings. Moreover, two studies reported poor discrimination measures for the PRISM-3 scoring system (AUC =0.667 [12] and 0.56 [33]), which might be due to specific conditions of their study sample (e.g., children receiving extracorporeal support for respiratory failure).

**Table 5:** Published evaluation studies of different versions of the two models in PICU

Study	Country	Sample Size(N)	Male Gender (%)	Age Mean ± SD Md (IQR)	Mortality Rate (%)	DX.	Score Mean ± SD Md (IQR)	AUC (95% CI)	Number of ICU	H-L *P*-value	Outcome^a^	SMR (95% CI)	LOS Mean ± SD Md (IQR)
[14]	ITALY	11,109	75%	68 (55–80) m	3.9%	Case-mix	PIM-3: 6 (5–8)	PIM-3:0.88 (0.86–0.89)	17 units	P:0.21	P	PIM-3:0.98 (0.89–1.08)	6.1 ± 15.5 2 (1–6)
[10]	Australia, New Zealand Ireland, UK	53,112	NA	NA	NA	Case-mix	NA	PIM-3:0.88 (0.88–0.89)	60 units	*p* > 0.95	P	PIM-3:1 (0.99–1.01)	NA
[15]	Australia New Zealand	26,966	NA	NA	4.3%	Case-mix	NA	PRISM-3:0.93 (0.92–0.94)	10 units	*P* < 0.001	P	PRISM-3:0.77 (0.72–0.82)	NA
[16]	Australia New Zealand	4403	55.5%	2.1 (0.6–6.6) m	5.2%	Invasive infection	NA	PIM-3:0.77 (0.730–0.81)	NA	NA	P	NA	4.72 ± 7.47
[17]	Poland	303	56.7%	Md:15.9	6.6%	Case-mix	NA	PRISM-3:0.78 (0.67–0.89)	NA	P: 0.21	P	PRISM-3:0.95 (0.68–1.23)	8.4 (2.0)
[18]	Portuguese	556	60%	65 (1–17) m	5%	Case-mix	PRISM-3: 5(0–46)	PRISM-3:0.92 (0.86–0.97)	Single	p: 0.282	P	PRISM-3:0.94 (0.60–1.28)	3 (0–155)
[8]	Poland	12,040	NA	NA	3.40%	Case-mix	NA	PRISM-3:0.90 (0.89–92)	7 units	NA	P	PRISM-3:0.87 (0.79–096)	NA
[19]	Greek	300	64.6%	49.93 ± 54.26 m	11.1%	Case mix	NA	PRISM-3: 0.89 (0.82–0.96)	Single	P:0.989	P	PRISM-3:0.99 (0.67–1.43)	8.85 ± 23.28
[20]	UK	10,197	57%	16.8 (2.4–79.2) m	6.2%	Case-mix	NA	PRISM-3: 0.82 (0.80–0.84)	18 units	*p* < 0.001	p	NA	Md:46.2
[21]	Belgium Netherlands Canada	1428	NA	1.44 (0.3–6.4) Yr.	4%	Case-mix	PRISM-3:8 (4–13) PIM-3: −3.5 (−4.4, −2.4)	PRISM-3:0.92 (0.91–0.92) PIM-3:0.89 (0.89–0.89)	3 units	P:0.04 P:0.5	p	NA	68 ± 4.8
[22]	Austria	398	55.8%	29.6 (3.8–105.6) m	13.6%	sepsis	(NA)	PRISM-3: 0.75 (0.68–0.75) PIM-3: 0.76 (0.68–0.76)	single	NA	P	NA NA	NA
[23]	Egypt	123	64.2%	5 (1–15) Yr.	20%	Case-mix	PRISM-3:36 (NA)	PRISM-3:0.91 (0.85–0.98)	Single	P: 0.65	p	NA	Mdn:5
[24]	Egypt	135	64.1%	26.4 ± 20.8 m	40%	Case-mix	PRISM-3: 6.02 ± 5.48	PRISM-3:0.875	Single	NA	P	NA	NA
[25]	Egypt	100	58%	8 (4–36) m	17%	Case-mix	(NA)	PRISM-3: 0.98 (0.96–1.0) PIM-3:0.97 (0.87–0.99)	single	*p* < 0.001 *p* < 0.001	H	PRISM-3: 2.11 PIM-3: 2.44	7(4,11)
[26]	India	723	59.1%	26.85 m	14.8%	Case-mix	NA	PRISM-3: (x > 0.86)	Single	P:0.395	P	PRISM-3: 1.01 (0.82–1.20)	NA
[12]	India	350	56.2%	12 (4–60) m	39.4%	Case-mix	NA	PRISM-3:0.66 (0.60–0.72) PIM-3:0.72 (0.67–0.78)	Single	P:0.747 P:0.059	P	PRISM-3:.90 (1.06–1.09) PIM-3:1.09 (0.91–1.27)	5(2–9)
[27]	Pakistan	370	64%	19 ± 25.8 m	23.8%	Case-mix	PRISM-3: 14 ± 7.30	PRISM-3:0.88 (0.84–0.92)	Single	P:0.244	P	NA	3 ± 1
[28]	Thailand	588	53.2%	37.9 (9.8–105) m	13.6%	Case-mix	PRISM-3: 4(0.8–9)	PRISM-3:0.845	single	NA	P	PRISM-3:0.98 (0.77–1.19)	3.5 (2–7.2)
[29]	Korea	503	59%	4.8 ± 4.6 Yr.	19.9%	Case-mix	NA	PRISM-3: 0.77 (0.73–0.81) PIM- 3: 0.82 (0.79–0.85)	Single	P:0.498 P:0.333	P	PRISM-3:NA PIM-3:1.1 (0.91–1.3)	17.1 ± 34.5
[30]	Korea	1710	59.2%	1.5 (0.3–7.8) Yr.	8.47%	Case-mix	NA	PIM-3:0.76 (0.72–0.80)	Single	0.313	P	NA	3 (1–8)
[31]	China	852	60.8%	6.5 (2–21) m	12.5%	Case-mix	PRISM-3: 11 ± 13.94	PRISM-3:0.72 (0.67–0.78)	single	p: 0.51	P	PRISM-3:1.14 (0.93–1.36)	8(4–15)
[32]	China	1109	NA	NA	10.4%	Case-mix	NA	PRISM-3: 0.82 (0.78–0.86)	single	p: 0.61	H	NA	NA
[2]	USA	3490	49%	16.4 (6.2–50.4) m	6.8%	Pulmonary hypertension	NA	PRISM-3:0.71 NA	143 units	NA	P	NA	NA
[33]	USA	178	NA	29 day-17Yr.	26%	Respiratory failure	Center1:19 (12–26) Center2:15 (11–21) Center3:9 (6–21)	PRISM-3:0.56 (0.46–0.66)	3 units	NA	P	NA	27(14–40]) 39(19–63) 25 (13–54)
[34]	Brazil	359	55%	31 (11–94) m	15%	Case-mix	PRISM-3: 15 (8–21)	PRISM-3:0.76 (0–69–0-83)	Single	p : 0.11	P	NA	5 (3–10)
[35]	Brazil	237	56%	12 (1–144) m	39%	Case-mix	PRISM-3:19 (9–42)	PRISM-3: 0.72 (0.66–0.79)	Single	P: 0.5	p	PRISM-3:1 (0.80–1.20)	Mean:7.5 Md:7.0
[36]	Argentina	6602	56.1%	20 (5–74) m	8%	Case-mix	NA	PIM-3:0.83 (0.82–0.85)	59 units	*P* < 0.001	P	PIM-3:1.3 (1.2–1.42)	5 (2–10)
[37]	Iran	221	57%	30 ± 5.85 m	21.3%	Case-mix	NA	PRISM-3:80.3	single	P:0.80	P	NA	NA
[38]	Iran	221	54%	29.85 ± 35.07 m	9.05%	Case-mix	PRISM-3: 14.2 ± 9.57	PRISM-3:0.89 (0.83–0.96)	single	P:0.161	P	PRISM-3:1.05 (NA)	5.16 ± 4.03
[39]	Iran	90	73.3%	93.6 (40.4146.7) m	17.8%	Case-mix	PRISM-3: 10.8 ± 5.1 PIM-3: 1.97 ± 1.30	PRISM-3:0.77 (0.71,0.83) PIM-3:0.82 (0.76,0.87)	single	P:0.79 P:0.93	P	NA NA	3.65 ± 3.95
[40]	Iran	365	60.2%	49 m	10.4%	Case-mix	PIM-3:1.45 ± (NA)	PIM-3:0.711 (0.63–0.80)	single	*P* < 0.001	P	PIM-3:7.18 (NA)	NA
THIS study	Iran	2446	5 6%	4.21 (0.66–24.1) m	16.1%	Case-mix	PIM-3: 2 ± 2.9 PRISM-3: 6 ± 6.5	PRISM-3:0.82 (0.80–0.85) PIM-3:0.74 (0.71–0.77)	6 units	*P* < 0.001 P:0.014	P&H	PRISM:1.34 (1.19–1.49) PIM:1.37 (1.21–1.52)	8(4–16)

Outcome^a^:P- PICU mortality, H- hospital mortality

In some studies, the Hosmer-Lemeshow test was used to evaluate the (lack of) concordance between observed versus predicted outcomes of the PIM-3 scoring system, which resulted in significant *p*-values (*P* = 0.003, *P* = < 0.001) [12, 41]. The PIM-3 performance was also evaluated in 49 PICUs in Argentina with 6602 patients aged between 1 month and 16 years and observed mortality rate was 8% (531/6602), whereas the predicted mortality by PIM-3 was 6.16% (407 deaths), moreover, the Hosmer-Lemeshow test showed disagreement between the predicted and observed mortality rates (χ2 = 135.63; *P* < 0.001) [36], supporting our result and Sankar and Wolfler studies [14, 41].

In our study the PRISM-3 model was well-calibrated, which is in line with findings provided by similar studies [12, 18, 19, 23, 27, 29, 32, 34, 35, 42]. However, there were also contradicting results showing poor calibration of these scoring systems. Aside from differences in the populations and selected sub-populations, this can also be due to the characteristics of the Hosmer-Lemeshow test as it is sensitive to the sample size (with larger sample size it tends to reject the null-hypothesis of agreement between the predicted and expected probabilities of the event) and cutoff points.

In several studies, it has been reported that a higher risk of mortality is associated with mechanical ventilation [2, 10, 23, 34]. The multivariable analysis of the Balkin et al. study showed that the ventilation support had the highest odds ratio among all covariates (OR: 2.1, 95% CI: 1.7–2.6), which is in line with our findings (*P* < 0.001) (11). This result was also confirmed by other studies, indicating the higher mortality rate for the patients admitted to the ICU with a higher number of organ failures [32, 35]. Also the prospective study in a pediatric oncology intensive care unit demonstrated that there is a significant relationship between mortality rate and diagnosis, the number of organ failures and ventilation support (*P* = 0.03, *P* < 0.001, *P* < 0.001, respectively) [23]. The presence of high urea and high creatinine, which often reflect low cardiac output or shock, suggests that renal function is an important prognostic indicator of mortality [2, 32].

### Strengths and limitations

We conducted the analysis in a large heterogeneous multicenter cohort. In addition, we used a comprehensive battery of performance measures and conducted a rigorous internal validation using bootstrapping [43–45]. There are also some limitations in the present study which are important to mention: First, the original scoring systems were based on the worst value for each variable in the first 24 h, whereas in the current investigation, measures were obtained during the first hour of admission. However, by fitting the logistic regression model based on the scores ameliorates this limitation. Second, due to the retrospective study in some cases we did not have all the key variable required to calculate the scores. However, we used imputation to cope with the missing values. Third, although we considered all types of disease in our study, many patients with heart disease are directed to heart hospitals and are not in our cohort, which hence contains a limited proportion of heart patients. In this sense the cohort is not representative of those subgroups of critically ill patients. Future studies are needed for developing these models in other populations and for externally validating these models.

## Conclusions

The prediction model based on PRISM-3 had superior predictive performance of that based on PIM-3 in discrimination, calibration, and accuracy of predicted probabilities. Further large validation studies are needed to consolidate these findings.

## Data Availability

The datasets used and/or analyzed during the current study available from the corresponding author on reasonable request.
